# Timely Surgical Intervention Leads to Better Sustained Coverage after Reconstructive Hip Surgery in Patients with Cerebral Palsy

**DOI:** 10.3390/children11030272

**Published:** 2024-02-21

**Authors:** Renée Anne van Stralen, Dagmar Raymond Jacques Kempink, Alexandra Frederika Titulaer, Denise Eygendaal, Max Reijman, Jaap Johannes Tolk

**Affiliations:** 1 Department of Orthopedics and Sports Medicine, Erasmus Medical Center Rotterdam—Sophia Children’s Hospital, Doctor Molewaterplein 40, 3015 GD Rotterdam, The Netherlands; d.kempink@erasmusmc.nl (D.R.J.K.); d.eygendaal@erasmusmc.nl (D.E.); m.reijman@erasmusmc.nl (M.R.); j.tolk@erasmusmc.nl (J.J.T.); 2 Department of Orthopedics, Leiden University Medical Centre, Albinusdreef 2, 2333 ZA Leiden, The Netherlands; 3 Department of Rehabilitation Medicine, Rijndam Revalidatie, 3015 CN Rotterdam, The Netherlands; stitulaer@rijndam.nl

**Keywords:** hip surveillance, cerebral palsy, orthopedic surgery

## Abstract

Background: In up to 45–90% of non-ambulatory patients with cerebral palsy (CP), progressive hip migration can be observed. The goal of this study was to determine whether the implementation of a national hip surveillance guideline affected the outcome of hip reconstructions. Methods: We reviewed 48 primary hip reconstructions at a median follow-up of 4.4 years. Surgical outcome was evaluated based on complication rates and radiographic evaluation postoperatively and at follow-up. Radiographic measurements included the migration percentage (MP), head–shaft angle and acetabular index. The impact of preoperative MP, postoperative MP, tone management, Gross Motor Function Classification System (GMFCS) classification and age on MP at follow-up were examined using a mixed model analysis. Results: A decrease in preoperative MP was noted, from a median of 75.0% (2014) to 39.0% (2020). Lower preoperative MP showed a significant correlation to lower MP postoperatively (*p* = 0.012). Postoperative MP was a significant independent predictor of a lower MP at follow-up (*p* = 0.002). Conclusions: This study shows an improvement in the timing of hip reconstruction in patients with CP after implementation of the hip surveillance guideline. A reduction in preoperative MP resulted in improved postoperative outcomes. A lower postoperative MP was the most important predictor for sustained containment of the hip.

## 1. Introduction

Cerebral palsy (CP) is a leading cause of physical impairment and disability in children. It affects 2–4 children per 1000 live births worldwide [1,2,3,4,5,6,7]. Progressive hip displacement is a common orthopedic finding in patients with cerebral palsy. In population-based studies, 45–90% of all non-ambulatory patients with CP develop hip (sub-)luxation [8,9,10,11,12,13]. Untreated hip migration can progress to a hip dislocation which can become painful and affect sitting, quality of life and functioning [11,14,15]. Using outcome measures such as the Caregiver Priorities and Child Health Index of Life with Disabilities (CPCHILD), it has been shown that severe hip displacement significantly reduces health-related quality of life in children with CP [16] and that reconstructive hip surgery shows significant improvements in CPCHILD scores [17]. Routine hip surveillance is warranted, and a timely referral to a specialized pediatric orthopedic service recommended [5,8,11,18,19]. The first national hip surveillance model is called the Cerebral Palsy Follow-up Program (CPUP) and was established in Sweden in 1994. The results from the first 20 years were published in 2014 and showed a significant decrease in the incidence of hip dislocation after initiating the surveillance program (*p* < 0.001) [9]. After the introduction of the CPUP, Australia [19,20], British Columbia [21] and the American Academy of Cerebral Palsy and Developmental Medicine (AACPDM) [22] introduced guidelines on hip surveillance as well. The Dutch guideline on hip surveillance in children with cerebral palsy was published in 2018 [23] and our practice changed accordingly. In the Dutch hip surveillance program, the rehabilitation consultant is the person conducting hip surveillance and requesting serial radiographs according to a standardized protocol. Before the introduction of this protocol, there was less awareness and children did not always get the appropriate radiographs. This led to a later moment of referral to a specialized orthopedic service (for instance when children became symptomatic); therefore, an indication for surgery was often made at a later age or with a higher preoperative migration. Part of the hip surveillance protocol is now that children get referred to an orthopedic service when the radiograph shows an MP of ≥30%. This means that children get referred much earlier and, therefore, that the surgeon can consider reconstructive surgery at an earlier time and with a lower preoperative MP.

There is some literature available on factors influencing the surgical outcome after hip reconstruction in children with cerebral palsy. Rutz et al. concluded that the preoperative MP was the most influential risk factor affecting the postoperative outcome in their cohort of 168 hip reconstructions, therefore emphasizing the need for timely intervention [24]. Minaie et al. analyzed 38 failed hip reconstructions in a total cohort of 291 hip reconstructions. Their analysis showed that an age below 6 years at the time of surgery significantly increased the failure rate, as did a preoperative migration percentage over 70% [25]. Next to that, it has been suggested that the Gross Motor Function Classification System (GMFCS) and age at the time of surgery may be risk factors leading to recurring instability after hip reconstructive surgery [26,27,28]. Considering that age and pre-operative MP are primary modifiable factors that influence surgical outcome, timely referral and surgical intervention is advocated to optimize treatment results.

Our primary research question was whether there was a change in surgical indication (lower MP at surgery) over time and whether this change had an impact on surgical outcome. Our secondary research questions were to determine which factors influenced surgical outcome directly postoperatively and at the final follow-up.

## 2. Methods

This retrospective study reviews the records of all CP patients that underwent reconstructive hip surgery between 2014 and 2020 in Erasmus MC—Sophia Children’s Hospital.

### 2.1. Patient Selection

**Figure 1:**
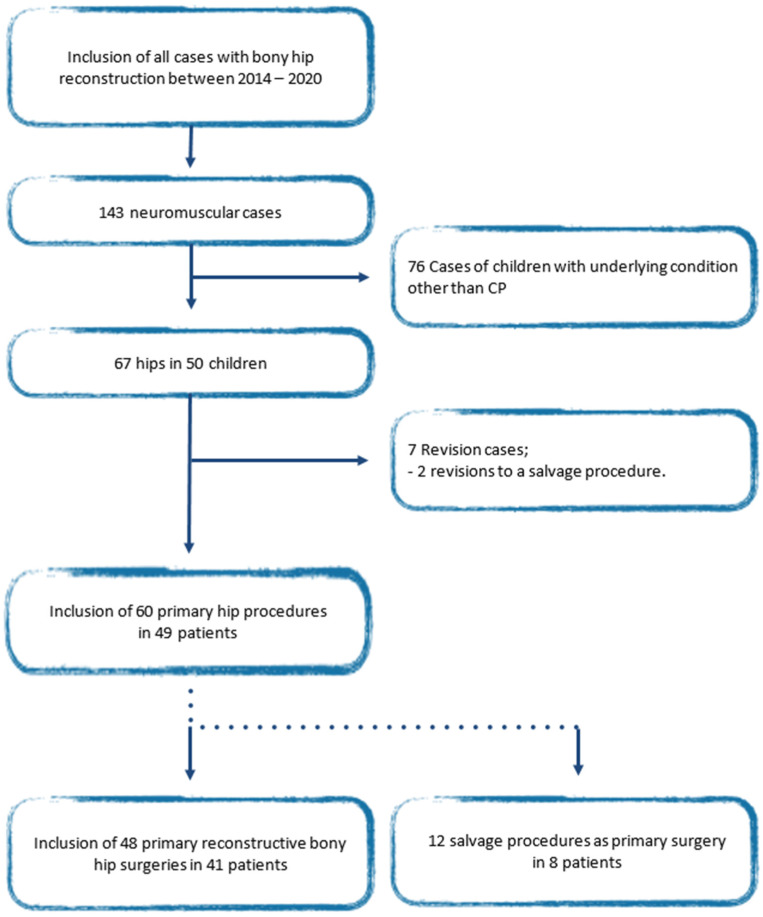
Flow chart of patient selection.

Between 2014 and 2020, 60 primary cases were performed in 49 patients with cerebral palsy. In 12 cases (8 patients) a salvage procedure was performed. A total of 48 primary reconstructive surgeries in 41 patients met the inclusion criteria. Reasons for inclusion and exclusion are specified in Figure 1.

Patients undergoing primary reconstructive surgery were included in all analyses. Patients undergoing salvage procedures could only be included in the analyses using the Melbourne CP hip classification system.

### 2.2. Data Extraction

After obtaining institutional board approval from Erasmus MC (MEC-2022-0619, 11 October 2022), we reviewed medical records for all consecutive patients diagnosed with neuromuscular disorders who underwent primary reconstructive hip surgery at our institution between 2014 and 2020. We included all patients with CP that had an adequate radiographic follow-up. Adequate radiographic follow-up was defined as at least one pre-operative and one postoperative radiograph. Patients with underlying neuromuscular disorders other than CP were excluded. Additionally, patients undergoing revision procedures were excluded. Salvage procedures were included in sub-analyses.

We included each hip as a unique case in the analysis. For each patient, a medical record review was performed by one author (RvS). Patient-specific information and surgical and radiographic data were collected. Patient age at time of surgery, sex, GMFCS level, tone management, type of CP (spastic vs. dyskinetic) and the extent of CP involvement were recorded. GMFCS level was determined according to the revised classification tool by Palisano et al. [29]. Surgical details that were recorded included: duration in minutes, estimated blood loss in milliliters, type of intervention (pelvic or femoral osteotomy or both), single or double surgeon and choice of implant. For all included patients, AP pelvis radiographs were evaluated. The radiographs that were included were preoperative radiographs, the first postoperative radiograph at six weeks postoperatively and the one at the last available follow-up. Radiographic measurements were performed using Vue PACS (2022 Koninklijke Philips NV, Best, Amsterdam, The Netherlands) and the person performing all radiographic measurements was blinded to the date of surgery. Reimer’s migration percentage (MP) is a radiographic measurement that quantifies hip displacement [30]. An MP of ≥30% is considered “at risk” for subluxation, and a migration percentage ≥50% is considered at risk for dislocation [14,31]. The head–shaft angle (HSA) describes the relationship between the neck and femoral head and was measured according to Southwick [32,33,34]. Secondary acetabular dysplasia, occurring as a consequence of coxa valga and hip migration, is assessed by measuring the acetabular index (AI) in degrees [35]. Next to that, preoperative radiographs and radiographs at the last available follow-up were classified according to the Melbourne classification of hip disease in cerebral palsy [36]. The Melbourne Cerebral Palsy Hip Classification System is based on the following gross morphologic features: integrity of Shenton’s arch; shape of the femoral head; shape of the acetabulum; and pelvic obliquity. The classification system ranges from Grade I (normal hip) to VI (Salvage surgery) [36].

### 2.3. Statistical Analysis

All data analyses were performed using IBM^®^ SPSS software, version 24.0 (SPSS Inc., Chicago, IL, USA). Significance was defined as *p* < 0.05. A Kolmogorov–Smirnov and Shapiro–Wilk test were used to test for normal distribution. Age at surgery, blood loss, preoperative HSA, postoperative MP, the available follow-up and MP at the final follow-up showed no normal distribution, so non-parametric tests were used for all analyses.

Continuous outcome parameters are presented as median and interquartile range (IQR) depending on data distribution and dichotomous measures as counts and percentages.

To answer the primary question, the change in indication over time was assessed using preoperative measurements of MP, HSA and Melbourne hip classification. A Spearman correlation coefficient was performed to assess for a linear relation between the year of surgery and MP, HSA and Melbourne Hip Classification. A scatter plot was formed to show changes over time in preoperative MP, postoperative MP and MP at the final follow-up. A fitted regression line was incorporated to depict the trend. The Melbourne cerebral palsy hip classification system was analyzed preoperatively and at the final follow-up, and a correlation analysis was performed to assess whether the Melbourne hip classification could be correlated to the year of surgery. Postoperative complication rates were described using percentages.

To answer the secondary research question, the influence of the preoperative MP on the MP at the final follow-up was analyzed using mixed models analysis, defining subjects by their study ID. GMFCS Classification, the use of tone management and single vs. double surgeon were included as factors in this analysis, and age at surgery, postoperative migration index and total available follow-up were included as covariates. Pre-operative MP was not included as a covariate due to a strong correlation with the direct postoperative MP. To correct for differences in follow-up time, we added follow-up in years in the model.

After the analysis, a post hoc power calculation was performed to assess whether the sample size was sufficient to calculate the detected differences.

## 3. Results

### 3.1. Patient Characteristics

The median age at the time of surgery was 8.0 years (IQR 7.0–9.6 years). Of these patients, 1 was GMFCS II, 2 patients were GMFCS III, 26 patients were GMFCS IV and 19 patients were GMFCS V. There were 15 patients that used tone management at the time of surgery; 14 patients had systemic baclofen; and 1 patient had a baclofen pump. No patients had had an SDR at the time of their hip reconstruction. In total, 34 cases underwent unilateral surgery, and 14 cases were bilateral hip reconstructions. The baseline characteristics of these cases are described in Table 1.

The median follow-up of all primary reconstructive cases was 4.4 years (IQR 3.6; 6.1 years), and 46 cases had a minimum follow-up of 2 years. In 11 hips (22.9%), a compression hip screw (Smith&Nephew, Memphis, TN, USA) was used, in 30 hips (62.5%), a pediatric hip plate (DePuy Synthes, Oberdorf, Switzerland) was used, in 6 cases (12.5%), a Locking cannulated blade was used (Orthopediatrics, Warsaw, IN, USA), and in 1 case, a Coventry infant hip screw (DePuy, Johnson&Johnson, Leeds, UK) was used to fixate the femoral osteotomy. The implant choice was based on implant availability and the surgeon’s preference. 

Furthermore, there were 12 cases of salvage procedures. Nine hips underwent a Schanz pelvic support osteotomy. There was one revision surgery where the patient underwent a valgus osteotomy and a Chiari pelvic osteotomy after a previous overcorrection. Additionally, there was one case where a proximal femoral osteotomy was combined with a shelf osteotomy.

### 3.2. Surgical Data

All cases underwent a Varus Derotational Osteotomy (VDRO). A total of 36 patients (75%) underwent adductor tenotomies as part of this procedure. An additional Dega-type pelvic osteotomy was performed in 30 cases.

**Figure 2:**
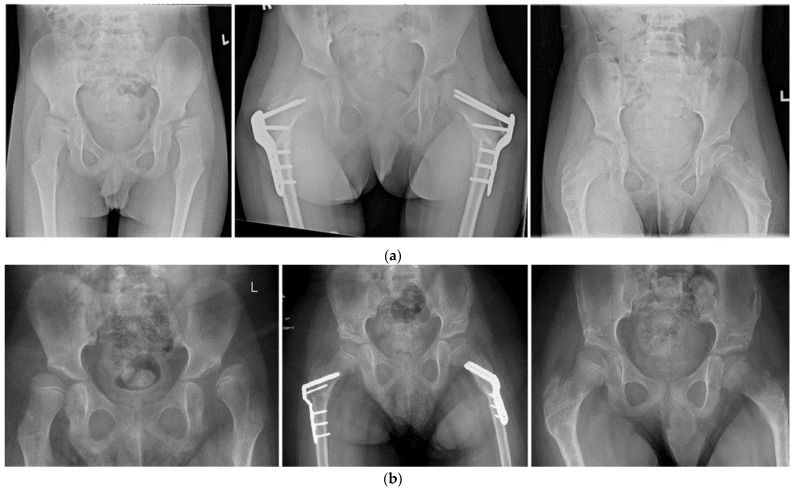
Case examples of hip reconstruction. (**a**) Case 1: Pediatric Hip Plate. Patient 1 is a boy with CP—GMFCS V spastic quadriplegia. The first radiograph is at age 6 years. At this time, he was scheduled for a bilateral VDRO †. The second radiograph shows postoperative results at 3 months postoperatively. The third radiograph shows the postoperative results at 1 year and 2 months postoperatively (which was 6 weeks after removal of metal). (**b**) Case 2: Locking Cannulated Blade Plate. Patient 2 is a boy with CP—GMFCS IV spastic diplegia. The first radiograph is at age 10 years. At this time, he was scheduled for a bilateral VDRO and bilateral Dega pelvic osteotomy. The second radiograph shows postoperative results at 6 months postoperatively. The third radiograph shows the postoperative results at 1 year and 2 months postoperatively and 6 weeks after removal of metal. † VDRO Varus derotation osteotomy.

The median surgical time (including anesthetic time) was 180.5 min (IQR 160.8–223.3 min). The median blood loss was 190.0 mL (IQR 100.0–250.0 mL). The surgical data are depicted in Table 2. Two examples of cases are shown in Figure 2.

### 3.3. Hip Development

For the complete cohort, including the salvage procedures (total population of 60 hips), the median preoperative MP was 62.5% (IQR 43.3–81.3%).

Including only the 48 hips that underwent primary reconstructive hip surgery, the median preoperative migration percentage (MP) was 57.5% (IQR 40.5–74.0%). Postoperatively, this MP decreased to a median of 11.0% (IQR 0.0–24.8%). At the final follow-up (after a median of 4.4 years), the median MP showed a slight increase to 23.0% (IQR 13.0–42.3%) (Table 3).

#### 3.3.1. Head–Shaft Angle Stratified by Year of Surgery

The median preoperative head–shaft angle was 165.0 degrees (IQR 157.3–169.0 degrees). The median postoperative head–shaft angle was 121.5 degrees (IQR 114.0–135.3 degrees), and the median head–shaft angle at the last available follow-up was 140.1 degrees (IQR 114.0; 153.6 degrees). (Table 3) We found no significant correlation between the year of surgery and pre-operative HSA (*p* = 0.647), postoperative HSA (*p* = 0.363) or HSA at last FU (*p* = 0.809).

#### 3.3.2. Migration Percentage Stratified by Year of Surgery

**Figure 3:**
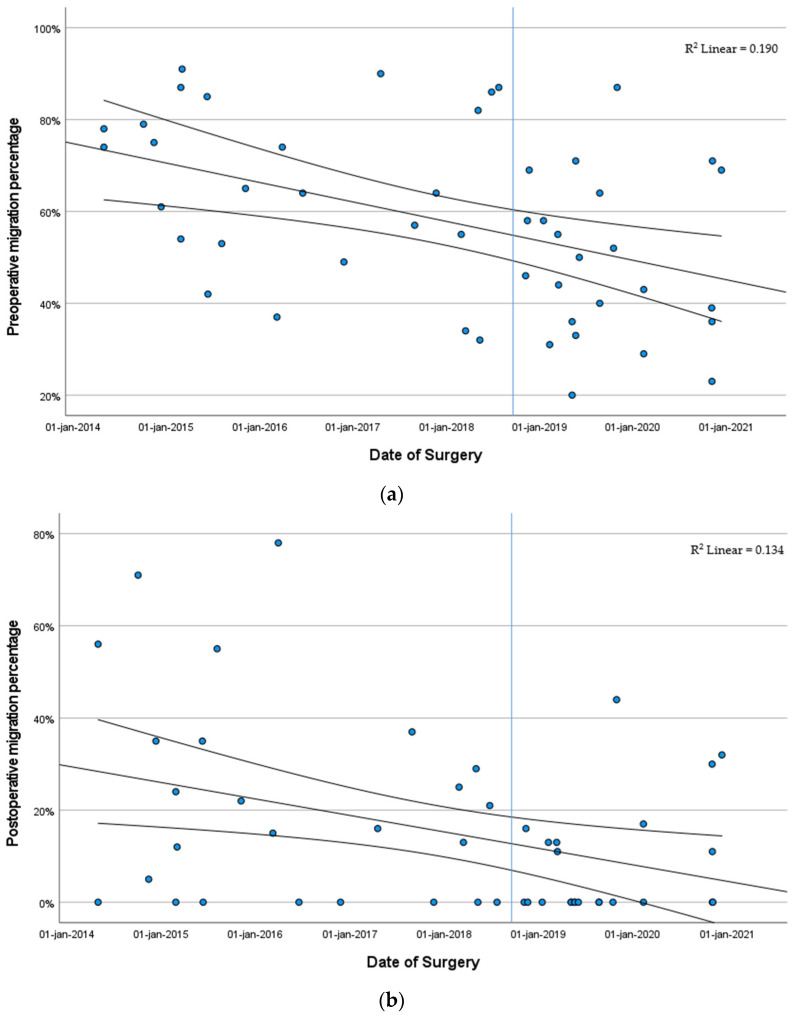

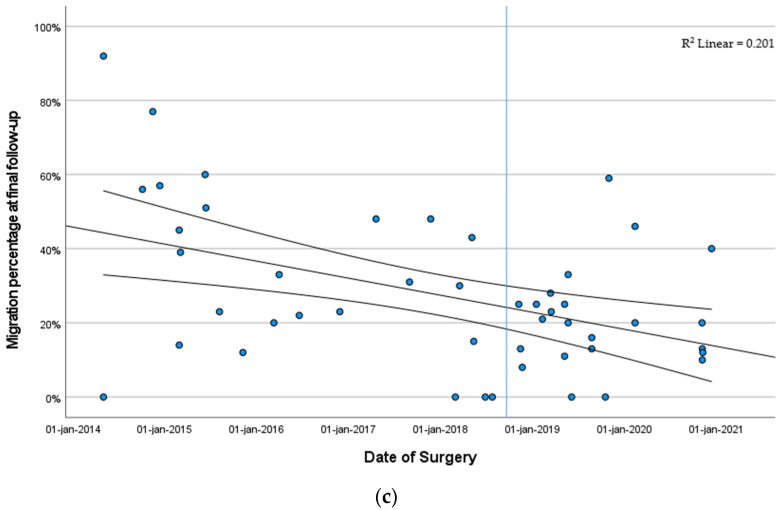
Migration percentages. (**a**) Date of surgery and preoperative migration percentage. Each point in the figure represents a hip reconstruction case. The lines shown depict the fit line and the 95% confidence interval. The vertical blue line depicts the formalization of the guideline for hip surveillance. (**b**) Date of surgery and migration percentage postoperatively. Each point in the figure represents a hip reconstruction case. The lines shown depict the fit line and the 95% confidence interval. The vertical blue line depicts the formalization of the guideline for hip surveillance. (**c**) Date of surgery and migration percentage at the final follow-up. Each point in the figure represents a hip reconstruction case. The lines shown depict the fit line and the 95% confidence interval. The vertical blue line depicts the formalization of the guideline for hip surveillance.

Looking at the median preoperative MP at the time of surgery in the complete cohort, we found a steady decline from an MP of 75.0% in 2014 (IQR 67.5–78.5%) to 39.0% (IQR 29.0–69.0%) in patients who underwent surgery in 2020. In the population that underwent primary reconstructive surgery, the median preoperative MP declined from 75.0% (IQR 67.5–78.5%) to 43.0% (IQR 32.5–64.0%) This decline in preoperative MP over time is visualized in Figure 3a. There was a significant correlation between the year of surgery and preoperative MP (*p* < 0.001).

For postoperative MP, a gradual decline was observed as well. In 2014, the median postoperative MP was 35.0% (IQR 2.5–63.5%) (*n* = 5), whereas the median postoperative MP was 11.0% (IQR 0.0–30.0%) in 2020 (*n* = 7) (Figure 3b). There was a significant negative correlation between the year of surgery and postoperative MP (*p* = 0.036).

Looking at the MP at the time of the final follow-up, there was a similar decline from 57.0% (IQR 28.0–84.5%) in patients that underwent surgery in 2014 to 20.0% (IQR 12.0; 40.0%) in patients that underwent surgery in 2020 (Figure 3c). There was a significant correlation between the year of surgery and MP at the final follow-up (*p* = 0.014).

A post hoc power analysis was performed, looking at a decrease from 75.0% MP to 39.0% in the current study population. In order to have a 90% chance of detecting this difference in the primary outcome as significant at the 5% level, four patients would be required in this retrospective study. As such, the current power is sufficient to detect this difference. 

#### 3.3.3. Risk Factors Impacting Hip Development

There is a significant correlation between preoperative MP and postoperative MP (correlation coefficient 0.359, *p* = 0.012). A mixed models analysis on factors potentially influencing the MP at the time of the final follow-up showed a significantly lower MP at the time of the final follow-up with a lower postoperative MP (*p* = 0.002) (Table 4). The maximum available follow-up had no significant relationship to MP at the final follow-up (*p* = 0.319).

The mixed models analysis shows a significant relationship where the use of tone management was associated with a higher migration percentage at the final follow-up (*p* = 0.032).

### 3.4. Melbourne Cerebral Palsy Hip Classification System

Preoperatively, the median grade for hips classified according to the Melbourne Cerebral Palsy Hip Classification System was IV (IQR grade IV–IV). At the final follow-up, the median grade was III (IQR grade III–IV) (Table 3). A correlation analysis showed a significant negative correlation between the year of surgery and the preoperative Melbourne hip classification score (correlation coefficient = −0.300, *p* = 0.038) and a significant negative correlation between the year of surgery and the Melbourne hip classification at the time of the last available follow-up (correlation coefficient = −0.448, *p* = 0.001).

### 3.5. Postoperative Complications

Out of a total of 48 cases, 21 cases had postoperative complications. Early postoperative complications consisted of failure of fixation (*n* = 9), under-correction requiring surgical revision (*n* = 2), wound infection (*n* = 1), pressure sores in the cast (*n* = 1), pressure sore on the heel (*n* = 1), increasing spasticity requiring additional treatment (*n* = 1) and a subtrochanteric fracture (*n* = 1). Late postoperative complications consisted of rebound valgus requiring additional surgery between 2.5 and 3.1 years after the index surgery (*n* = 3) and avascular necrosis of the femoral head (*n* = 2). In addition to that, 2 cases had an MP of ≥60% at the final follow-up. This means that in 5 cases of the 48 cases (10.4%), reconstructive hip surgery failed.

There was no statistically significant difference in the rates of complications between the year of surgery (*p* = 0.250).

## 4. Discussion

This study shows that the start of a dedicated multidisciplinary CP clinic with the implementation of a formal hip surveillance program led to earlier referrals and a subsequent decline in MP at the time of indicating patients for reconstructive hip surgery. The current study also shows that a lower preoperative MP correlates with a lower postoperative MP. A mixed model analysis showed that MP directly postoperatively is the most important predictor of sustained hip containment at the final follow-up. These results emphasize the need for timely surgery. Sustained containment is a good measure of surgical success and implies the limited need for revision surgery in the future. Given the impact of this type of surgery on children, their families and their mobility, this is valuable information for the considerations surrounding the timing of reconstructive hip surgery.

There is relatively limited information available about the factors influencing the long-term outcome after reconstructive hip surgery. Herndon et al. described a retrospective cohort of 48 hips in 32 children to determine the best time for surgical reconstruction. They found that the better the hip was located preoperatively, the better the postoperative results that were obtained. The best long-term results were achieved after a better postoperative result [37]. This corresponds well with the cohort Rutz et al. describe, where they conclude that preoperative MP is the most important factor to impact surgical outcome [24]. These results are similar to the results we describe in this series. Zhang et al. described a cohort of 58 reconstructive hip surgeries in GMFCS IV and V patients [38]. They found a percentage of 26% patients that required revision surgery or had an MP ≥ 60%. In our cohort, this was 10.4%. The mean follow-up was slightly longer in their study (62.5 months versus 4.4 years in our study), which means that the longer follow-up could contribute to the higher number of failures in their cohort [38].

The complication rates were not excessive or atypical for this type of surgical intervention and are comparable to the complication rates described by Rutz et al. [24].

The use of tone management did not safeguard against progressive hip migration after hip reconstruction. On the contrary, the mixed models analysis showed a significant relationship where the use of tone management was associated with a higher migration percentage at the final follow-up. This might reflect that the children using tone management were relatively more affected and non-ambulant.

Some limitations have to be addressed. The results of the secondary analysis on hip migration at the final follow-up might have been influenced by a relatively shorter follow-up for the more recent cases. Ideally a study would describe follow-up until skeletal maturity to be able to assess the need for secondary surgery in all cases. Next to that, the most recent cases seem to have a relatively better outcome, but the follow-up is shorter in these cases. This means these cases might have a lower chance of rebound valgus and progressive migration, leading to a bias towards a relatively better outcome for the more recent cases. Further studies with a longer follow-up are necessary to assess the longevity of these postoperative results. A second limitation is the fact that this study only discusses radiographic parameters. Pain, quality of life and patient-reported outcome measures are not taken into consideration as postoperative results, even though they are essential aspects of the surgical outcome. DiFazio et al. examined health-related quality of life (HRQOL) in 38 non-ambulatory patients with CP [39]. They concluded that reconstructive hip surgery reliably improves HRQOL and that their findings could aid in surgical decision making [39]. It can be expected that the cohort described in this study would show a similar relationship between preoperative and postoperative MP and preoperative and postoperative HRQoL as described by DiFazio et al. [39].

Analyses in this paper mainly focused on the timing of surgery in relation to the surgical outcome. Besides changes in this timing, several other alterations regarding perioperative care and organization of care have been gradually implemented. This led to earlier referrals and a better communication with referring centers and, therefore, the ability to consider surgery at a relatively earlier migration percentage. Next to that, postoperative management was improved by involving a nurse practitioner and involvement of a pediatrician and pediatric neurologists. We are convinced that all these changes had a positive influence on the final surgical outcome as well, but the quantification of each of them separately is beyond the scope of the analyses performed in this manuscript.

## 5. Conclusions

This research shows an improvement in the timing of surgical hip reconstruction in children with CP. A lower MP at indication was associated with a better postoperative outcome. A lower MP postoperatively was shown to be the most important factor in sustained hip coverage at follow-up. This study emphasizes the need for a guideline-based treatment protocol and stresses the need for a dedicated multidisciplinary neuromuscular unit for this vulnerable population. 

## Figures and Tables

**Table 1:** Demographics of the total population of bony hip reconstructions (*n* = 48) in children with CP ^†^ (2014–2020).

Median Age (IQR *) at Time of Surgery, Years	8.0 (IQR 7.0; 9.6)
Male: Female	29: 19
GMFCS ^‡^ level, *n* (%)	
I	0
II	1 (2.1%)
III	2 (4.2%)
IV	26 (54.2%)
V	19 (39.6)
CP type, *n* (%)	
Spastic	45 (93.8%)
Dystonic	3 (6.3%)
CP extent, *n* (%)	
Hemiplegia	1 (2.1%)
Diplegia	14 (29.2%)
Quadriplegia	33 (68.8%)
Tone management, *n* (%)	
Missing/unknown	1 (2.1%)
None	32 (66.7%)
Oral Baclofen	14 (29.2%)
Baclofen pump	1 (2.1%)
Median follow-up of all primary reconstructive hip surgeries, years (IQR)	4.4 (3.6; 6.1)
Median follow-up of all primary hip surgeries, including salvage procedures, years (IQR)	4.2 (2.8; 5.3)

^†^ CP: cerebral palsy, * IQR: interquartile range, ^‡^ GMFCS: Gross Motor Function Classification System.

**Table 2:** Surgical details.

Surgical Data	*n* (%)
Varus derotation osteotomy	48 hips (100%)
+ adductor tenotomies	36 hips (75%)
+ pelvic osteotomy	30 hips (62.5%)
Dega pelvic osteotomy	28 hips (58.3%)
Pemberton type osteotomy	1 hip (2.1%)
Salter type osteotomy	1 hip (2.1%)
Median blood loss, ml (IQR °)	190 mL (IQR 100; 250)
Median surgical time, minutes (IQR)	178 min (IQR 161; 222)

° IQR Interquartile range. + means additional procedures performed in the same operative sitting.

**Table 3:** Radiological parameters.

	Preoperative	Postoperative	Final Follow-Up
MP, %	57.5	11.0	23.0
	(40.5; 74.0)	(0.0; 24.8)	(13.0; 42.3)
HSA, degrees	165.0	121.5	140.1
	(157.3; 169.0)	(114.0; 135.3)	(114.0; 153.6)
AI, degrees	28.5	21.0	*
	(22.0; 32.0)	(15.5; 27.5)	
MCPHCS, grades	IV		III
	(IV; IV)		(III; IV)

Data are presented as median and IQR between parentheses: IQR: interquartile range, MP: migration percentage, HSA: head–shaft angle, AI: acetabular index, MCPHCS: Melbourne Cerebral Palsy Hip Classification System. * At final follow-up, the triradiate cartilage was closed in almost all patients, making it less reliable to measure an acetabular index.

**Table 4:** Mixed models analysis on predictors for the MP ^†^ at the time of final follow-up.

	Beta	95% Confidence Interval	*p* Value
Age at time of surgery	2.13	−0.87 to 5.12	*p* = 0.157
GMFCS ^‡^ II-III vs. IV-V	−2.03	−25.10 to 20.91	*p* = 0.855
Single vs. Double surgeon	−8.58	−20.28 to 3.11	*p* = 0.144
Tone management	−14.10	−26.90 to −1.30	*p* = 0.032
Postoperative MP	0.46	0.17 to 0.75	*p* = 0.002
Total available follow-up	1.43	−1.46 to 4.32	*p* = 0.319

^†^ MP: migration percentage. ^‡^ GMFCS: Gross Motor Function Classification System.

## Data Availability

The data presented in this study are available on request from the corresponding author. The data are not publicly available due to security and confidentiality concerns.

## References

[1] Arneson C.L., Durkin M.S., Benedict R.E., Kirby R.S., Yeargin-Allsopp M., Van Naarden Braun K., Doernberg N.S. (2009). Prevalence of cerebral palsy: Autism and Developmental Disabilities Monitoring Network, three sites, United States, 2004. Disabil. Health J..

[2] Bhasin T.K., Brocksen S., Avchen R.N., Van Naarden Braun K. (2006). Prevalence of four developmental disabilities among children aged 8 years—Metropolitan Atlanta Developmental Disabilities Surveillance Program, 1996 and 2000. MMWR Surveill. Summ..

[3] Jonsson U., Eek M.N., Sunnerhagen K.S., Himmelmann K. (2019). Cerebral palsy prevalence, subtypes, and associated impairments: A population-based comparison study of adults and children. Dev. Med. Child. Neurol..

[4] Paneth N., Hong T., Korzeniewski S. (2006). The descriptive epidemiology of cerebral palsy. Clin. Perinatol..

[5] Surveillance of Cerebral Palsy in Europe (2000). Surveillance of cerebral palsy in Europe: A collaboration of cerebral palsy surveys and registers. Surveillance of Cerebral Palsy in Europe (SCPE). Dev. Med. Child. Neurol..

[6] Van Naarden Braun K., Doernberg N., Schieve L., Christensen D., Goodman A., Yeargin-Allsopp M. (2016). Birth Prevalence of Cerebral Palsy: A Population-Based Study. Pediatrics.

[7] Winter S., Autry A., Boyle C., Yeargin-Allsopp M. (2002). Trends in the prevalence of cerebral palsy in a population-based study. Pediatrics.

[8] Connelly A., Flett P., Graham H.K., Oates J. (2009). Hip surveillance in Tasmanian children with cerebral palsy. J. Paediatr. Child. Health.

[9] Hagglund G., Alriksson-Schmidt A., Lauge-Pedersen H., Rodby-Bousquet E., Wagner P., Westbom L. (2014). Prevention of dislocation of the hip in children with cerebral palsy: 20-year results of a population-based prevention programme. Bone Jt. J..

[10] Lonstein J.E., Beck K. (1986). Hip dislocation and subluxation in cerebral palsy. J. Pediatr. Orthop..

[11] Soo B., Howard J.J., Boyd R.N., Reid S.M., Lanigan A., Wolfe R., Reddihough D., Graham H.K. (2006). Hip displacement in cerebral palsy. J. Bone Jt. Surg. Am..

[12] Terjesen T. (2012). The natural history of hip development in cerebral palsy. Dev. Med. Child. Neurol..

[13] Wordie S.J., Bugler K.E., Bessell P.R., Robb J.E., Gaston M.S. (2021). Hip displacement in children with cerebral palsy. Bone Jt. J..

[14] Bagg M.R., Farber J., Miller F. (1993). Long-term follow-up of hip subluxation in cerebral palsy patients. J. Pediatr. Orthop..

[15] Pritchett J.W. (1983). The untreated unstable hip in severe cerebral palsy. Clin. Orthop. Relat. Res..

[16] Ramstad K., Jahnsen R.B., Terjesen T. (2017). Severe hip displacement reduces health-related quality of life in children with cerebral palsy. Acta Orthop..

[17] DiFazio R., Vessey J.A., Miller P., Van Nostrand K., Snyder B. (2016). Postoperative Complications After Hip Surgery in Patients With Cerebral Palsy: A Retrospective Matched Cohort Study. J. Pediatr. Orthop..

[18] Shrader M.W., Wimberly L., Thompson R. (2019). Hip Surveillance in Children With Cerebral Palsy. J. Am. Acad. Orthop. Surg..

[19] Wynter M., Gibson N., Willoughby K.L., Love S., Kentish M., Thomason P., Graham H.K., National Hip Surveillance Working G. (2015). Australian hip surveillance guidelines for children with cerebral palsy: 5-year review. Dev. Med. Child. Neurol..

[20] Wynter M., Gibson N., Kentish M., Love S., Thomason P., Kerr Graham H. (2011). The development of Australian Standards of Care for Hip Surveillance in Children with Cerebral Palsy: How did we reach consensus?. J. Pediatr. Rehabil. Med..

[21] British Columbia’s Consensus on Hip Surveillance for Children with Cerebral Palsy. https://www.childhealthbc.ca/sites/default/files/clinical_booket_hip_surveillance_march_2018.pdf.

[22] The American Academy of Cerebral Palsy and Developmental Medicine: Hip Surveillance Care Pathways. https://www.aacpdm.org/publications/care-pathways/hip-surveillance-in-cerebral-palsy.

[23] Spastische Cerebrale Parese BIJ Kinderen: Screening ter Preventie van Heupluxatie. https://richtlijnendatabase.nl/richtlijn/spastische_cerebrale_parese_bij_kinderen/behandeling_gericht_op_verbetering_van_mobiliteit/effect_van_orthopedisch_chirurgie_op_mobiliteit/screening_ter_preventie_van_heupluxatie.html.

[24] Rutz E., Vavken P., Camathias C., Haase C., Junemann S., Brunner R. (2015). Long-term results and outcome predictors in one-stage hip reconstruction in children with cerebral palsy. J. Bone Jt. Surg. Am..

[25] Minaie A., Gordon J.E., Schoenecker P., Hosseinzadeh P. (2022). Failure of Hip Reconstruction in Children With Cerebral Palsy: What Are the Risk Factors?. J. Pediatr. Orthop..

[26] Bayusentono S., Choi Y., Chung C.Y., Kwon S.S., Lee K.M., Park M.S. (2014). Recurrence of hip instability after reconstructive surgery in patients with cerebral palsy. J. Bone Jt. Surg. Am..

[27] Chang F.M., May A., Faulk L.W., Flynn K., Miller N.H., Rhodes J.T., Zhaoxing P., Novais E.N. (2018). Outcomes of Isolated Varus Derotational Osteotomy in Children With Cerebral Palsy Hip Dysplasia and Predictors of Resubluxation. J. Pediatr. Orthop..

[28] Terjesen T. (2017). To what extent can soft-tissue releases improve hip displacement in cerebral palsy?. Acta Orthop..

[29] Palisano R., Rosenbaum P., Walter S., Russell D., Wood E., Galuppi B. (1997). Development and reliability of a system to classify gross motor function in children with cerebral palsy. Dev. Med. Child. Neurol..

[30] Reimers J. (1980). The stability of the hip in children. A radiological study of the results of muscle surgery in cerebral palsy. Acta Orthop. Scand. Suppl..

[31] Pons C., Remy-Neris O., Medee B., Brochard S. (2013). Validity and reliability of radiological methods to assess proximal hip geometry in children with cerebral palsy: A systematic review. Dev. Med. Child. Neurol..

[32] Southwick W.O. (1967). Osteotomy through the lesser trochanter for slipped capital femoral epiphysis. J. Bone Jt. Surg. Am..

[33] Chougule S., Dabis J., Petrie A., Daly K., Gelfer Y. (2016). Is head-shaft angle a valuable continuous risk factor for hip migration in cerebral palsy?. J. Child. Orthop..

[34] Hermanson M., Hagglund G., Riad J., Wagner P. (2015). Head-shaft angle is a risk factor for hip displacement in children with cerebral palsy. Acta Orthop..

[35] Tonnis D. (1976). Normal values of the hip joint for the evaluation of X-rays in children and adults. Clin. Orthop. Relat. Res..

[36] Robin J., Graham H.K., Baker R., Selber P., Simpson P., Symons S., Thomason P. (2009). A classification system for hip disease in cerebral palsy. Dev. Med. Child. Neurol..

[37] Herndon W.A., Bolano L., Sullivan J.A. (1992). Hip stabilization in severely involved cerebral palsy patients. J. Pediatr. Orthop..

[38] Zhang S., Wilson N.C., Mackey A.H., Stott N.S. (2014). Radiological outcome of reconstructive hip surgery in children with gross motor function classification system IV and V cerebral palsy. J. Pediatr. Orthop. B.

[39] DiFazio R., Shore B., Vessey J.A., Miller P.E., Snyder B.D. (2016). Effect of Hip Reconstructive Surgery on Health-Related Quality of Life of Non-Ambulatory Children with Cerebral Palsy. J. Bone Jt. Surg. Am..

